# Utility of endoscopic ultrasound-guided hepatic abscess drainage with contrast-enhanced harmonic imaging

**DOI:** 10.1055/a-2413-7672

**Published:** 2024-09-25

**Authors:** Noriyuki Onishi, Masahiro Itonaga, Masayuki Kitano

**Affiliations:** 1 13145 Second Department of Internal Medicine, Wakayama Medical University, Wakayama, Japan

Percutaneous transhepatic abscess drainage (PTAD) is the first-choice treatment for patients with a hepatic abscess. Recent reports suggest that endoscopic ultrasound-guided hepatic abscess drainage (EUS-HAD) is useful for treating hepatic abscesses in areas that are difficult to puncture with PTAD 1 2 3 . Although identification of the hepatic abscess is important for safe performance of EUS-HAD, the borderline between the hepatic abscess and hepatic parenchyma is sometimes unclear. Although contrast-enhanced harmonic imaging (CHI) is known to enhance the marginal border of a hepatic abscess during PTAD 4 5 , no reports have examined the utility of EUS-HAD with CHI. Here, we examined the utility of EUS-HAD with CHI when treating a hepatic abscess with a border that was unclear with the hepatic parenchyma.

A 76-year-old woman was admitted to our institution because of obstructive jaundice caused by pancreatic cancer. After biliary drainage had been performed, the patient developed a hepatic abscess ( Fig. 1 **a** ). We decided to treat this using EUS-HAD because computed tomography (CT) revealed ascites on the hepatic surface, making a percutaneous approach difficult ( Fig. 1 **b** ). Because fundamental B-mode EUS failed to clearly identify the border between the abscess and the hepatic parenchyma ( Fig. 2 **a** ), CHI was performed after intravenous injection of an ultrasound contrast agent. The borderline was then clear enough to determine the puncture line ( Fig. 2 **b** ). The hepatic abscess was punctured with a 19G EUS fine-needle aspiration (FNA) needle under CHI, and access to the abscess was confirmed after the aspiration of pus and injection of contrast medium ( Video 1 ). After a 0.025-inch guidewire had been inserted into the abscess via the needle, the puncture site was dilated with a 7-Fr bougie dilator. Finally, an endoscopic nasobiliary drainage tube was placed into the abscess.

**Fig. 1:**
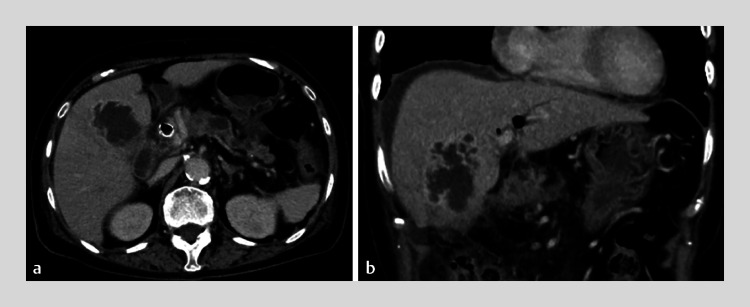
Contrast-enhanced computed tomography image showing: **a** the hepatic abscess in the right lobe of the liver; **b** ascites on the hepatic surface, which made a percutaneous approach difficult.

**Fig. 2:**
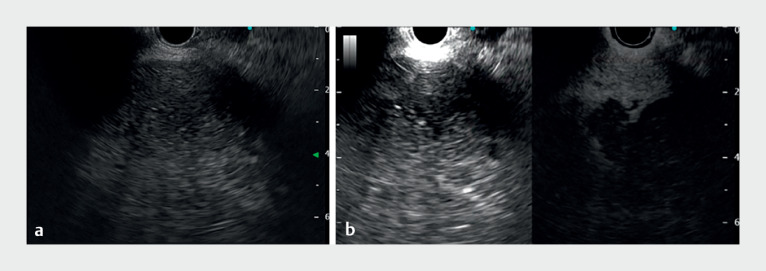
Endoscopic ultrasound (EUS) images using: **a** fundamental B-mode EUS showing a mosaic area within the hepatic parenchyma, within which the border of the hepatic abscess is unclear; **b** contrast-enhanced harmonic EUS after bolus injection of ultrasound contrast agent has enhanced the hepatic parenchyma but not the abscess, making it easy to visualize the border of the hepatic abscess.

Endoscopic ultrasound-guided hepatic abscess drainage with contrast-enhanced harmonic imaging.Video 1

In conclusion, CHI may be a method of safely performing EUS-HAD because it clarifies the border between the hepatic abscess and the parenchyma.

Endoscopy_UCTN_Code_TTT_1AS_2AD

## Notes

Endoscopy E-Videos https://eref.thieme.de/e-videos
*E-Videos* is an open access online section of the journal *Endoscopy* , reporting on interesting cases and new techniques in gastroenterological endoscopy. All papers include a high-quality video and are published with a Creative Commons CC-BY license. Endoscopy E-Videos qualify for HINARI discounts and waivers and eligibility is automatically checked during the submission process. We grant 100% waivers to articles whose corresponding authors are based in Group A countries and 50% waivers to those who are based in Group B countries as classified by Research4Life (see: https://www.research4life.org/access/eligibility/ ). This section has its own submission website at https://mc.manuscriptcentral.com/e-videos .

## References

[1] Seewald S Imazu H Omar S EUS-guided drainage of hepatic abscess Gastrointest Endosc 2005 61 495 498 15758937 10.1016/s0016-5107(04)02848-2

[2] Noh SH Park DH Kim YR EUS-guided drainage of hepatic abscesses not accessible to percutaneous drainage (with videos) Gastrointest Endosc 2010 71 1314 1319 20400078 10.1016/j.gie.2009.12.045

[3] Ogura T Masuda D Saori O Clinical outcome of endoscopic ultrasound-guided liver abscess drainage using self-expandable covered metallic stent (with video) Dig Dis Sci 2016 61 303 308 26254774 10.1007/s10620-015-3841-3

[4] Morita M Ogawa C Omura A The efficacy of Sonazoid-enhanced ultrasonography in decision-making for liver abscess treatment Intern Med 2020 59 471 477 32062622 10.2169/internalmedicine.2510-18 PMC7056389

[5] Catalano O Sandomenico F Raso MM Low mechanical index contrast-enhanced sonographic findings of pyogenic hepatic abscesses AJR Am J Roentgenol 2004 182 447 450 14736679 10.2214/ajr.182.2.1820447

